# Cisplatin-Loaded Polybutylcyanoacrylate Nanoparticles with Improved Properties as an Anticancer Agent

**DOI:** 10.3390/ijms20071531

**Published:** 2019-03-27

**Authors:** Seyed Ebrahim Alavi, Sitah Muflih Al Harthi, Hasan Ebrahimi Shahmabadi, Azim Akbarzadeh

**Affiliations:** 1 Department of Pilot Nanobiotechnology, Pasteur Institute of Iran, Tehran 009821, Iran; s.ebrahimalavi@gmail.com; 2 Department of Pharmaceutical Science, College of Pharmacy, Shaqra University, Aldwadmi 11911, Saudi A0096611, Saudi Arabia; s_alharthi@su.edu.sa; 3 Department of Microbiology, School of Medicine, Rafsanjan University of Medical Sciences, Rafsanjan 009834, Iran

**Keywords:** cisplatin, polybutylcyanoacrylate, polyethylene glycol, temperature, lung cancer, histological study

## Abstract

This study aims to improve the cytotoxicity and potency of cisplatin-loaded polybutylcyanoacrylate (PBCA) nanoparticles (NPs) for the treatment of lung cancer through the modulation of temperature and polyethylene glycol (PEG) concentration as effective factors affecting the NPs’ properties. The NPs were synthesized using an anionic polymerization method and were characterized in terms of size, drug loading efficiency, drug release profile, cytotoxicity effects, drug efficacy, and drug side effects. In this regard, dynamic light scattering (DLS), scanning electron microscopy (SEM), 3-(4,5-dimethylthiazol-2-yl)-2,5-diphenyl tetrazolium bromide (MTT) methods, and hematoxylin and eosin (H&E) staining were used. The results showed that the size and the drug loading efficiency of the synthesized spherical NPs were 355–386 nm and 14–19%, respectively. Also, the drug release profile showed a controlled and slow drug release pattern with approximately 10% drug release over 48 h. In addition, the NPs significantly increased the cytotoxicity of the cisplatin in vitro environment by approximately 2 times and enhanced the therapeutic effects of the drug in vivo environment by increasing the survival time of lung-cancer-bearing mice by 20% compared to the standard drug receiver group. Also, the nanoformulation decreased the drug toxicity in an in vivo environment. According to the results, increasing the temperature and PEG concentration improved the properties of the drug loading efficiency, drug release profile, and cytotoxicity effect of drug-loaded NPs. Consequently, the synthesized formulation increased the survival of tumor-bearing mice and simultaneously decreased the cisplatin toxicity effects. In conclusion, the prepared nanoformulation can be considered a promising candidate for further evaluation for possible therapeutic use in the treatment of lung cancer.

## 1. Introduction

Cisplatin is an anticancer drug and is widely used in the treatment of solid tumors, such as testicular, cervical, ovarian, head and neck, and lung cancer [1,2,3]. Cisplatin binds to the N7-positions of guanosine bases in deoxyribonucleic acid (DNA) molecules and disrupts DNA replication, leading to cell death [4]. However, cisplatin therapy has some drawbacks, including the increase of tumor resistance to cisplatin and the development of several severe side effects [5]. Using higher doses of cisplatin can overcome tumor resistance; however, this increases the drug’s side effects, owing to the non-target-specificity of cisplatin [6]. Therefore, targeted delivery of cisplatin can significantly improve its efficacy and simultaneously decrease the side effects of cisplatin. In this regard, nanodrug delivery systems such as polybutylcyanoacrylate (PBCA) nanoparticles (NPs) are considered an appropriate strategy and have received considerable attention as they increase drugs’ specificity and reduce their side effects [7].

PBCA NPs have been widely used for drug delivery applications as they are biodegradable and can change the biological distribution of therapeutic compounds in the human body. Moreover, these NPs are synthesized and purified easily and are able to overcome multidrug resistance (MDR) [8]. PBCA NPs are usually synthesized using an anionic polymerization method [9,10,11] due to it providing a high rate of polymerization and particles with small size [12]. Also, there are some important factors affecting the NPs’ properties, including pH, monomer concentration, temperature, and stabilizer [13]. The NP polymerization is initiated by nucleophilic attack to the β-carbon of butylcyanoacrylate followed by the production of carbanion, which reacts with further monomer to prepare oligomeric chains which are nucleated, resulting in NP formation. The concentration of OH^−^, as the major initiating nucleophile, varies with pH. At high pH (high OH^−^ concentration), the polymerization rate is rapid; therefore, discrete particles are formed, resulting in the direct polymerization of monomer droplets and the generation of an amorphous polymer mass. As the pH decreases, the polymerization rate is reduced, and the NPs are formed [14]. For monomer concentrations below 1% (*v*/*v*), the polydispersity index is high; however, it is rapidly decreased with increasing monomer concentration [14]. Also, at low temperatures, NP agglomeration occurs due to the entrapment of un-reacted monomer within the matrix of NPs [15]. In addition, in the absence of a stabilizer, the NPs are formed but eventually will form agglomerates [15]. PBCA NPs have been widely used as a drug carrier for the treatment of various malignancies, including lung cancer [1,8,16].

Lung cancer, as a health threat, is the leading cause of cancer-related death worldwide. Despite the existence of various strategies such as chemotherapy and radiotherapy for the treatment of lung cancer, the prognosis of the disease is still poor [17]. The application of current chemotherapy is limited due to the lack of target specificity, recurrence, and superficial increase in longevity of patients. Moreover, oral and intravenous (IV) administrations of anti-cancer drugs cause various issues, such as drug molecule degradation in the stomach pH, drug molecule alterations during the process of liver metabolism, and the lack of specificity in the conventional treatment methods, which cause toxicity and side effects [18].

In the present study, the effect of various temperatures and polyethylene glycol (PEG) concentrations on the properties of cisplatin-loaded PBCA NPs was evaluated and modulated to improve the therapeutic efficacy in in vitro and in vivo environments. PEG is an NP stabilizer and has been commonly used in various studies [19,20,21,22]. It can decrease the toxicity effects of nanoformulations [23]. Also, PEG incorporation into the NP structure improves their water solubility and inhibits their aggregation. Moreover, PEGylation decreases serum protein adsorption onto NPs and reduces their capture rate by the reticuloendothelial system [23]. Herein, temperature and PEG concentration were modulated to prepare cisplatin-loaded PBCA NPs with improved properties, and their efficacy was evaluated for the treatment of lung cancer. To this end, 3-(4,5-dimethylthiazol-2-yl)-2,5-diphenyltetrazolium bromide (MTT), photon correlation spectroscopy (PCS), atomic absorption spectroscopy (AAS), spectrophotometry, scanning electron microscopy (SEM), and Hematoxylin and Eosin (H&E) staining methods were used.

## 2. Results and Discussion

### 2.1. Preparation of Cisplatin-Loaded PBCA NPs

Cisplatin-loaded PBCA NPs were successfully synthesized by the dropwise addition of BCA monomer into the polymerization medium. Polymerization was performed in the presence of dextran 70 KDa as a stabilizer. The temperature was set by a thermometer; 10 min after monomer addition, the color of the medium was changed from colorless to milky, implying monomer polymerization. All the prepared batches (A1, A2, A3, and A4) were the same in appearance; however, more aggregates were observed in the beakers containing the NPs prepared at 25 °C, indicating that the yield of the NPs prepared at this temperature was lower than that of those synthesized at 65 °C. The agglomeration might be due to the un-reacted monomers which were entrapped in the NPs.

### 2.2. Size, Size Distribution, and Zeta Potential of NPs

Behan et al. reported that the temperature of 65 °C was the optimum for synthesizing PBCA NPs. They observed that the rate of primary particle production was increased as the temperature was raised [15]. Moreover, previous research showed that PEGylation increased the size of NPs [24].

The results of the present study showed that all the NP batches (A1, A2, A3, and A4) had negative zeta potential. The highest and the lowest zeta potential, with values of −7 ± 0.8 mV and −11 ± 1.4 mV, corresponded to the A3 and A2 NPs, respectively. Regardless of the charge type (positive or negative), the charged NPs were stable in aqueous solutions with low ionic strength, due to repulsive forces [25]. Also, it was observed that PEGylated NPs with 1% *w*/*v* of PEG, either blank or containing the drug, had less negative zeta potential than did those with 0.25% *w*/*v* of PEG. It has been found that PEGylation increases NPs’ surface charge [26] and drug loading efficiency [27]. In the current study, the zeta potential of NPs containing the drug was increased as cisplatin has positive charge [28]. The results of one study showed that PBCA prepared with anionic polymerization had a glass transition temperature (T_g_) at 55 °C [29]. Also, Behan et al. reported that particles prepared at above T_g_ were soft and coalesced into a semisolid mass in hot water, resulting in the production of larger particles. In the current study, the temperature used for preparing PBCA NPs (65 °C) was above T_g_, leading to having larger particles in batches A2 and A4 (Table 1). Moreover, the hydrophilic nature of PEG might help to increase the hydrodynamic diameter of NPs as each ethylene glycol unit absorbs two water molecules [2]. This effect was also observed in PEGylated proteins [30]. Overall, it seems that the effects of temperature rising are more efficient than the effects of increasing PEG concentration on increasing the NPs’ size. Also, larger NPs had more positive zeta potential, which could be the result of their higher cisplatin content (drug loading efficiency) as cisplatin is a positively charged molecule.

### 2.3. Evaluation of the NP Morphology

**Figure 1:**
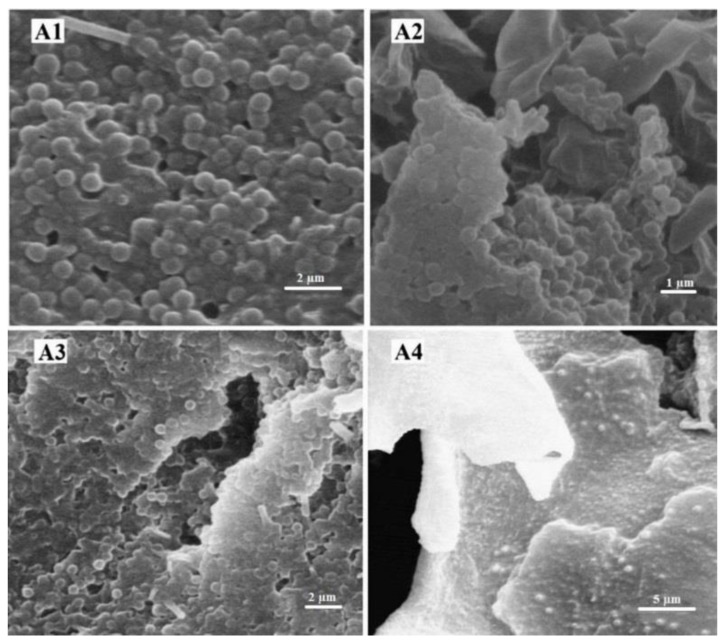
SEM images of the four different batches of cisplatin-loaded PBCA NPs. (**A1**) (0.25% *w*/*v* PEG concentration, room temperature), (**A2**) (0.25% *w*/*v* PEG concentration, 65 °C), (**A3**) (1.0% *w*/*v* PEG concentration, room temperature), and (**A4**) (1.0% *w*/*v* PEG concentration, 65 °C).

The obtained electron micrograph of four batches of the NPs indicated the formation of monodispersed nanospheres with a relatively narrow distribution. Regardless of the effects of temperature and PEG concentration, the particles had a smooth surface without considerable surface fracture or pitting even at higher magnifications (Figure 1).

### 2.4. Evaluation of Cisplatin Loading Efficiency

Research has shown that a PEG coating on nanocarriers increases the drug loading efficiency [27]. Also, it has been found that the drug loading efficiency is directly associated with the size of NPs [31,32].

The results of the present study showed that the drug loading efficiency was increased as the size of the NPs was increased. The results indicated that the drug loading efficiencies of the A1, A2, A3, and A4 batches were 14%, 17%, 15%, and 19%, respectively. In other words, 14%, 17%, 15%, and 19% of the primary drug used were loaded into the NPs. Also, the NPs prepared at 65 °C had higher drug content compared to those synthesized at 25 °C. This is in accordance with results from Zhaparova et al. [33], indicating that rising temperature helped the reaction to progress, resulting in an increase in the yield of polymerization. In addition, the result of the present study showed that the drug loading efficiency was increased by increasing the PEG concentration (14% and 15% for A1 and A3, respectively).

### 2.5. Drug Release Study

In the current study, a dialysis membrane technique was used for the release study. Drug release from nanocarriers determines drug therapeutic effects [34]. Increasing the PEG concentration decreases the drug release from nanocarriers [35]. A controlled drug delivery system which slowly releases a drug at the therapeutic concentration at the treatment site increases the drug efficacy and decreases the drug side effects. Also, this system removes the short bursts of overmedication seen in conventional drug delivery systems [36]. Moreover, using biodegradable materials for nanoparticle construction, such as PEG and dextran, helps sustain drug release at the target site for a few days or even weeks after injection [37].

**Figure 2:**
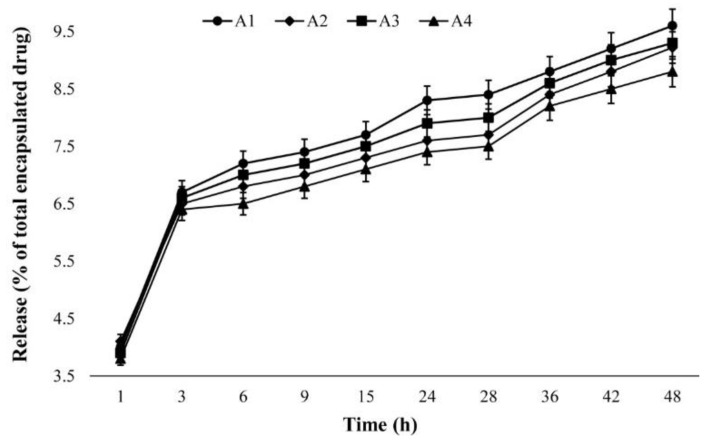
The cumulative cisplatin release percentage versus time for the A1 (0.25% *w*/*v* PEG concentration, room temperature), A2 (0.25% *w*/*v* PEG concentration, 65 °C), A3 (1.0% *w*/*v* PEG concentration, room temperature), and A4 (1.0% *w*/*v* PEG concentration, 65 °C) batches synthesized using an anionic polymerization technique at different time intervals. The results are expressed as mean ± 5% values from three independent experiments.

The results of the present study showed a slow controlled drug release pattern for all batches, in which approximately 10% of the loaded drug was released over 48 h (Figure 2). The highest amount of drug release occurred in the first hour of the study (a burst release), where approximately 4% of the loaded drug was released. This might be due to the release of adsorbed cisplatin on the NPs. Overall, the release curve demonstrated a sharp rise in the first three hours of the study that remained steady for the duration of the experiment. The drug release patterns for all batches were approximately similar. Also, the results showed that the amount of released drug from the A1 nanoformulation (9.6%) was greater than that from the A4 nanoformulation (8.8%). This might be due to the effect of particle size in the release profile, in which the A1 nanoformulation, as the smallest particle, released the higher amount of cisplatin. Particles with smaller size have higher surface area which causes more initial burst release and, consequently, a greater amount of total drug release [38].

### 2.6. Cytotoxicity of Cisplatin-Loaded NPs

The results of various studies have shown that nanocarriers enhance drug cytotoxicity [39,40]. Also, one study showed that docetaxel-loaded PEGylated NPs increased the cytotoxicity effects of the drug more than did docetaxel-loaded non-PEGylated NPs [41]. Moreover, the results of one study showed that PEGylation of a polystyrene nanocarrier increased its biocompatibility, resulting in facilitating their transport across cell membranes [42].

**Figure 3:**
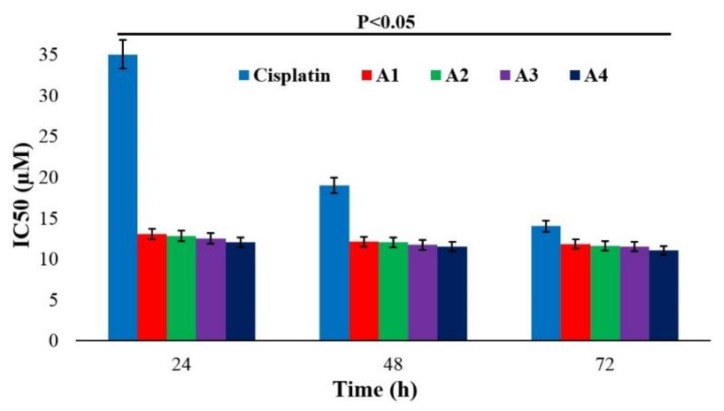
The cytotoxicity effects (IC_50_) of standard cisplatin and cisplatin-loaded PBCA NPs on the LLC1 cell line at different time intervals. The results are presented as mean ± 5% values from three independent tests. A1 (0.25% *w*/*v* PEG concentration, room temperature), A2 (0.25% *w*/*v* PEG concentration, 65 °C), A3 (1.0% *w*/*v* PEG concentration, room temperature), and A4 (1.0% *w*/*v* PEG concentration, 65 °C).

In the present study, the LCC1 cell line was used, and the non-toxic concentration of blank NPs was determined to be 30 µg/mL. The results showed that the cytotoxicity effects of all batches of nanoformulations containing cisplatin were significantly increased compared to the standard drug (*p* < 0.05). This could be due to slow and controlled drug release from the NPs at the therapeutic concentration. Also, the cytotoxicity potency of PBCA NPs was more considerable within the first 24 h (*p* < 0.05) compared to that of standard cisplatin. This effect was more considerable for the A4 batch, in which the cytotoxicity of cisplatin was increased by 2.9-fold due to the less negative surface charge and the larger size of the A4 NPs. Particles with less negative surface charge interact more with cells compared to those with more negative charge, owing to the negative charge of the cell membrane. Therefore, the chance of NPs binding to and penetrating cells is increased [43]. Also, research has shown that larger particles are more easily endocytosed than small particles, resulting in higher cellular cytotoxicity [44]. Furthermore, the cytotoxicity effects of all batches and the standard cisplatin formulation were increased by increasing the incubation time, in which the highest cytotoxicity effects were achieved after 72 h incubation. However, this increase was not significant for all batches (Figure 3). Overall, the PBCA NPs were shown to be completely potent in increasing the cytotoxicity effects of cisplatin, and consistency was observed between the drug loading efficiency and cytotoxicity effects.

### 2.7. Evaluation of the NPs’ Stability

Researchers have shown that PEGylation enhances NP stability due to the steric repulsion effects of tethered PEG strands [45]. Also, PEG can enhance the stability of drugs against enzymatic degradation, including proteases or nucleases, and decrease drug immunogenicity [46]. In addition, the results of previous studies showed that PBCA NPs have proper stability [8,47] as they are polymeric stable nanocarriers, owing to rigidity of their matrix/shell and their capability to maintain their structure for long period of time when used topically [48].

**Figure 4:**
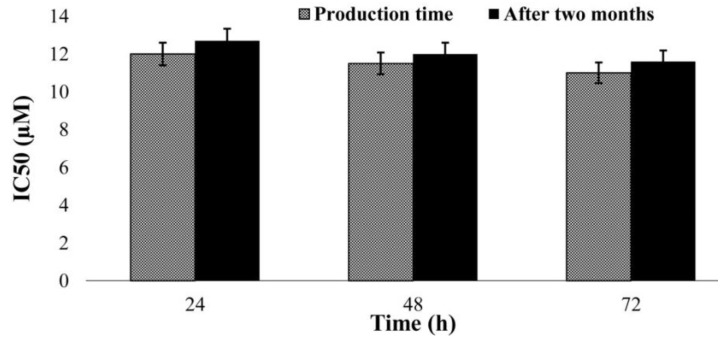
The cytotoxicity effects (IC_50_) of cisplatin-loaded PBCA NPs (A4; 1.0% *w*/*v* PEG concentration, 65 °C) on the LLC1 cell line in the first day after production and two months later. The results are presented as mean ± 5% values from three independent tests.

In the present study, the cytotoxicity effects of A4 NPs on LCC1 cell line were evaluated two months after synthesis and compared to those at the synthesis time. The results showed no considerable difference, indicating that the prepared NPs were sufficiently stable to preserve cisplatin potency (*p* < 0.05) (Figure 4).

### 2.8. In Vivo Antitumor Efficacy of the Formulations

The ability of nanocarriers to increase drug efficacy against lung cancer has been shown in various studies [49,50,51]. Zhang et al. [49] prepared a PLGA-based nanostructure loaded with doxorubicin and observed enhanced antitumor efficacy against a xenograft tumor model in BALB/c nude mice. In another study, Lv et al. [50] co-delivered doxorubicin and paclitaxel using a PEG–polypeptide nanocarrier for the treatment of non-small cell lung cancer. The results of this study showed enhanced therapeutic effect and reduced drug side effects. In addition, Choi et al. [51] synthesized inhalable self-assembled albumin NPs containing doxorubicin and evaluated their efficacy for the treatment of drug-resistant lung cancer. The results showed that the nanodrug was remarkably effective in reducing the tumor size compared to the standard drug.

In the present study, the antitumor efficacy of cisplatin-loaded PBCA NPs was evaluated through the establishment of a heterotopic model of lung cancer. Regarding the in vivo evaluation of antitumor efficacy, the A4 batch was selected due to it having the greatest cytotoxicity effects among all batches. The survival time was measured in the drug (standard and loaded drug) receiver mice and compared to that of the control group. The results showed that the survival time of the tumor-bearing mice group receiving the nanodrug was considerably increased compared to the survival times of the groups receiving the standard drug and PBS (as control group) 35 days after tumor cell transplantation (12 vs. 9 vs. 6 alive mice, respectively). In other words, the survival times of tumor-bearing mice and the standard drug receiver group were increased 50% and 25%, respectively, compared to the survival time of the control group.

**Figure 5:**
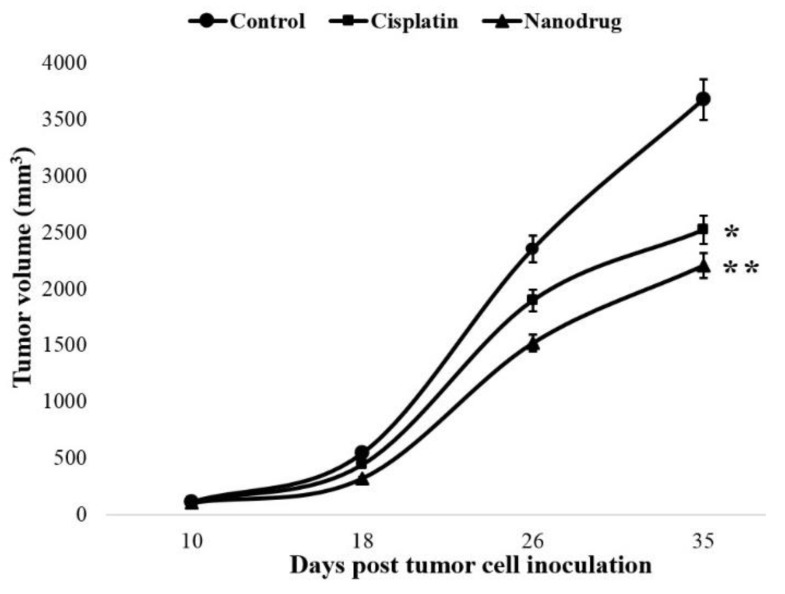
The changes of tumor volume in the standard and nanodrug receiver groups compared to the control group. Statistical significance: * *p* < 0.05, ** *p* < 0.01.

Also, the results of tumor volume measurement indicated that tumor volume was decreased significantly in both the drug and nanodrug receiver mice compared to the control group. However, this reduction was more noticeable in the nanodrug receiver mice (Figure 5). The tumor volume was decreased in the standard drug receiver mice by 32% 35 days after tumor cell inoculation, while this value for the nanodrug receiver mice was 40%. Furthermore, to evaluate and confirm the antitumor efficacy of the nanodrug, the tumor growth inhibition index (TGII) was measured. The results showed that the TGII was more increased in the nanodrug receiver group (40%) compared to in the animals received the standard drug (31%) 35 days after the tumor cell transplantation (Table 2).

**Figure 6:**
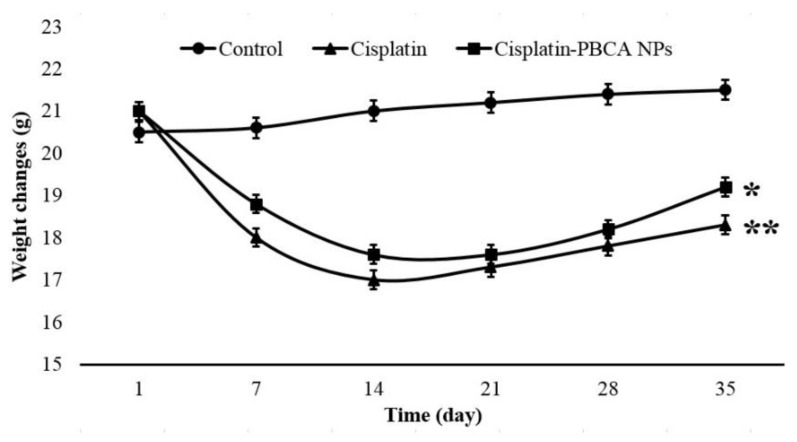
The weight changes in the lung-cancer-bearing mice treated with cisplatin and the nanodrug compared to the control group. The results are expressed as mean ± 5% of the values obtained from monitored mice in each group.

One of the well-known complications of cisplatin is cachexia [52]. During a period of 35 days, weight changes of the animals were measured, and the results showed that cisplatin caused a significant body weight loss in animals (*p* < 0.01). However, body weight loss in the nanodrug receiver mice was considerably less than that in standard drug group (*p* < 0.05), indicating the potency of NPs in decreasing the drug toxicity effects (Figure 6).

**Figure 7:**
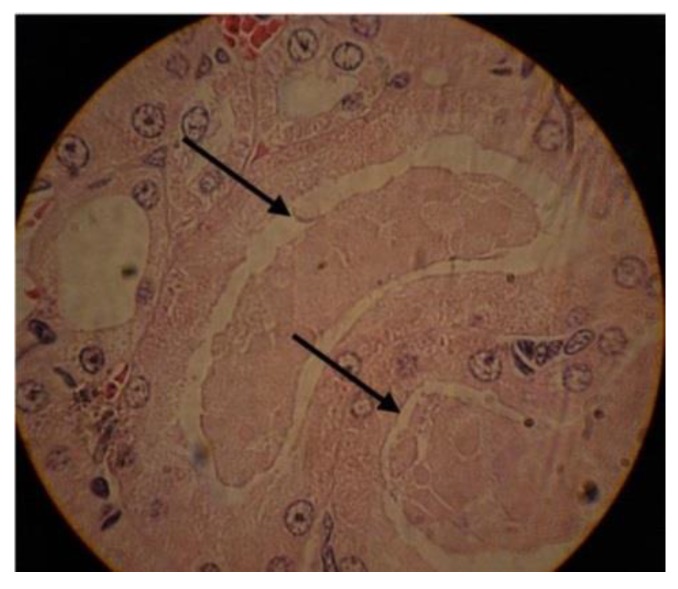
The hematoxylin and eosin (H&E) staining results of kidney tissue in cisplatin receiver mice. The arrows show the acute tubular necrosis (ATN) injury (×40).

In addition, the toxicity effects were evaluated using histopathological study and H&E staining. The results demonstrated that the nanodrug receiver mice had fewer histopathological lesions compared to the mice received the standard drug. This effect was more significant in the kidney, where acute tubular necrosis (ATN) was more prevalent in the cisplatin receiver mice compared to in the nanodrug receiver group (Figure 7).

Overall, the in vivo results of the present study were consistent with the in vitro results in that the nanodrug had higher anticancer potency compared to the standard drug.

## 3. Materials and Methods

### 3.1. Materials

Butylcyanoacrylate (BCA) monomer and dextran 70 KDa were purchased from Evobond^®^Tong Shen Enterprise Co., Ltd. (Taiwan) and Zhejiang Chemicals Import and Export Corporation (Hangzhou, China), respectively. Hydrogen chloride, MTT, phosphate-buffered saline (PBS), sodium hydroxide, mannitol, H&E, a dialysis bag (cut-off of 10,000 Da), and cisplatin were obtained from Sigma-Aldrich (St. Louis, MO, USA). Roswell Park Memorial Institute (RPMI)-1640 medium, penicillin/streptomycin antibiotic, and fetal bovine serum (FBS) were obtained from Gibco (Carlsbad, CA, USA). PEG2000 was purchased from Kimiagaran Emrooz Chemical Ind. (Arak, Iran). C57BL/6 mice and lung cancer cell line (LCC1) were supplied by Pasteur Institute of Iran, Tehran. All other materials were of analytical grade. Deionized water was used throughout the study.

### 3.2. Preparation of Cisplatin-Loaded PBCA NPs

Cisplatin-loaded PBCA NPs were synthesized according to the previous method [10] with some modifications. Briefly, 1.0% (*v*/*v*) BCA monomer was added to the polymerization medium containing 0.01 N chloridric acid, cisplatin (1.0 mg/mL), PEG2000 (0.25% and 1.0% (*w*/*v*)), and 2.0% (*w*/*v*) of dextran 70 KDa. The medium was stirred under two different conditions (500 RPM, 4 h, room temperature; and 500 RPM, 4 h, 65 °C). The pH was then adjusted to 7.0 using NaOH (0.1 N). Agitation was continued for 1 h to complete the polymerization process. Four batches of cisplatin-loaded NPs were prepared, namely, A1 (0.25% *w*/*v* PEG concentration, room temperature), A2 (0.25% *w*/*v* PEG concentration, 65 °C), A3 (1.0% *w*/*v* PEG concentration, room temperature), and A4 (1.0% *w*/*v* PEG concentration, 65 °C). Blank NPs were prepared according to the abovementioned method at PEG concentrations of 0.25% and 1.0% at room temperature and at 65 °C without adding the drug.

### 3.3. Size, Size Distribution, and Zeta Potential of NPs

The size, size distribution, and zeta potential of the NPs were determined using the PCS method. For this purpose, NP suspension was diluted in PBS, and their absorbance was calculated by a spectrophotometry method at 630 nm. The suspension was then introduced to a Zetasizer instrument (ZEN 3600, Malvern Instruments Ltd., Worcestershire, UK).

### 3.4. Evaluation of the NP Morphology

The morphology of the NPs was evaluated using scanning electron microscopy (SEM, ZEISS, Germany). The NP suspension was lyophilized (Edwards High Vacuum, Manor Royal, Crawley, Sussex, England) after the addition of mannitol (3.0% *w*/*v*). Mannitol is a cryoprotectant and prevents PBCA NPs from being damaged during the lyophilization process [53]. The lyophilized powder of NPs was then metalized with gold and visualized with an SEM instrument.

### 3.5. Evaluation of the Cisplatin Loading Efficiency

The drug loading efficiency was determined by the AAS method (HR-CS GFAAS) model ContrAA 700 (Analytik Jena, Germany) using H2PtCl6 (Sigma Aldrich, USA) as a standard. For this purpose, the nanodrug suspensions were centrifuged (15,000 RPM, 30 min, 4 °C) and their related supernatants were obtained. The drug concentration in the supernatants were then measured using AAS at the absorbance of 265 nm from three repeated experiments. Next, the drug loading efficiency was calculated using formula (1):

(1) $$Drug\;loading\;efficiency\;\left(\%\right)=\frac{initial\;drug\;concentration\;\left(mg\right)-drug\;concentration\;in\;supernatant\;\left(mg\right)}{initial\;drug\;concentration\;\left(mg\right)}\times 100.$$

### 3.6. Drug Release Study

Drug release studies were performed using a dialysis membrane technique [54]. For this purpose, suspensions of cisplatin-loaded NPs were centrifuged (15,000 RPM, 30 min, 4 °C) and their related pellets were obtained. The pellets containing 5 mg of cisplatin were then resuspended in 5 mL of fresh PBS and transferred into four individual dialysis bags; they were separately immersed into 50 mL of PBS as an acceptor medium and stirred (150 RPM, room temperature). Cisplatin’s solubility in PBS is 1.0 mg/mL, confirming the maintenance of sink conditions throughout the release experiments. At predetermined time intervals, 2 mL of PBS was collected and replaced with 2 mL of fresh PBS. The cisplatin concentrations in the collected samples were calculated using the AAS method. The cumulative drug release percentage versus time was estimated using formula (2), and the relative curve was plotted:

(2) $$Drug\;release\;\left(\%\right)=\frac{W_{release}}{W_{total}}\times 100$$

where W_total_ is the total amount of cisplatin in the NPs and W_release_ is the drug amount released from the NPs into the acceptor medium at the various times.

### 3.7. Cytotoxicity of Cisplatin-Loaded NPs

The cytotoxicity effects of cisplatin-loaded PBCA NPs were evaluated by MTT assay on the LCC1 cell line [55]. The cells were cultured in 96-well plates containing RPMI-1640 medium supplemented with 10% FBS and 1% penicillin/streptomycin antibiotics at the density of 10^4^ cells/well. After 24 h of incubation, the cells were attached onto the bottom of the wells. The media were then replaced with media containing the standard drug and cisplatin-loaded PBCA NPs at the drug concentrations of 0, 2, 4, 8, 16, 32, 64, 128, and 256 µM. After that, the plates were incubated for 24, 48, and 72 h (5% CO_2_, 37 °C). Later, the culture media were removed, 100 µL of MTT (0.5 mg/mL PBS) was added, and the plates were incubated for 3 h (5% CO2, 37 °C). Isopropanol was then replaced with MTT to dissolve the formazan crystals, and the absorbance was read at 540 nm using a microplate scanning spectrophotometer (ELISA reader; Organon Teknika, Boxtel, the Netherlands). Cell viability was calculated using formula (3) [56]:

(3) $$Cell\;viability\;\left(\%\right)=\frac{Int_{s}}{Int_{control}}\times 100$$

where Int_s_ is the absorbance of the cells treated with the standard drug and nanodrug, and Int_control_ is the absorbance of the cells treated with the medium only.

The half maximal inhibitory concentration (IC_50_) of the compounds was calculated using the statistical package Pharm-PCS software. All experiments were performed in triplicate.

### 3.8. Evaluation of the NPs’ Stability

To evaluate the NPs stability, the cytotoxicity effects of the A4 NPs were evaluated two months after their preparation on LCC1 cells using MTT assay. The results were compared to those for the first day of the NPs’ preparation.

### 3.9. In Vivo Antitumor Efficacy of the Formulations

In this study, C57BL/6 male mice at the age of 6–8 weeks (weight of 18–22 g) were used. The animals were housed under the condition of 25 ± 2 °C, 12 h light/12 h dark cycle, and relative humidity of 55 ± 5%. They had free access to standard food and water. The experiments were approved by the Animal Experimentation Ethics Committee of Pasteur Institute of Iran, Tehran. After one week, the mice were inoculated subcutaneously (right groin) with LLC1 cells (100 µL of PBS containing 5 × 10^5^ cells). One week later, they were randomly divided into three groups (*n* = 15) and received the standard drug, cisplatin-loaded PBCA NPs, or PBS. The mice received 1.5 mg/kg of cisplatin in the standard form or loaded onto a nanocarrier intraperitoneally at time intervals of 72 h and six times in total. The antitumor efficacy of cisplatin was evaluated in the different formulations by measuring the survival time of animals in the different groups. Tumor volume (mm^3^) was also determined at various time intervals using a external caliper and formula (4):

(4) $$Tumor\;volume=\left(length\times width^{2}\right)\times 0.5.$$

Furthermore, the tumor growth inhibition index (TGII) was calculated using formula (5):

(5) $$TGII=\frac{W_{c}-W_{exp}}{W_{c}}\times 100$$

where W_C_ and W_Exp_ are the mean tumor weight of the control mice group and the mean tumor weight of the treatment mice group, respectively. In addition, the toxicity of the formulations was assessed by measuring the weight changes of animals and histopathological evaluation of tumors, livers, and kidneys.

### 3.10. Histological Evaluation

Histological evaluation was performed through preparing successive sections of paraffin-embedded tissue and H&E staining. Briefly, three mice from each group were sacrificed 35 days after tumor cell inoculation and organ toxicity was assessed using a semiquantitative scoring system of 0 in the case of no toxicity symptoms, 1 as observing any slight change, and 2 for medium changes in organs.

### 3.11. Statistical Analysis

The results of the study were analyzed using SPSS version 15.0 software (SPSS Inc., Chicago, IL, USA), and *p* < 0.05 was considered significant.

## 4. Conclusions

The clinical application of cisplatin as an anticancer drug is limited due to its severe side effects. Controlled drug delivery systems, such as PBCA NPs, can increase the therapeutic efficacy of anticancer drugs and decrease their side effects. In the current study, cisplatin was loaded onto PBCA NPs, and the two important factors of temperature and PEG concentration were modulated to improve the properties of the nanoformulations. It was found that by increasing the temperature from 25 °C to 65 °C and the PEG concentration from 0.25% to 1.0%, the drug loading efficiency and the anticancer properties of cisplatin were increased. Also, the results showed that the synthesized formulation with 1.0% (*w*/*v*) PEG at the temperature of 65 °C (A4) had the highest effects of cytotoxicity, drug loading efficiency, and in vivo antitumor efficacy. Therefore, it can be considered as a promising therapeutic option for further evaluation for the treatment of lung cancer.

## Figures and Tables

**Table 1:** The size, size distribution, and zeta potential of various batches of cisplatin-loaded polybutylcyanoacrylate (PBCA) nanoparticles (NPs). A1 (0.25% *w*/*v* PEG concentration, room temperature), A2 (0.25% *w*/*v* PEG concentration, 65 °C), A3 (1.0% *w*/*v* PEG concentration, room temperature), and A4 (1.0% *w*/*v* PEG concentration, 65 °C).

	Properties	Size (nm)	Size Distribution	Zeta Potential (mV)
Batches of NPs				
A1		355.0 ± 32.0	0.44 ± 0.05	−10.0 ± 0.8
A2		382.0 ± 39.0	0.38 ± 0.07	−11.0 ± 0.4
A3		360.0 ± 40.0	0.25 ± 0.02	−7.0 ± 0.3
A4		386.0 ± 37.0	0.31 ± 0.02	−8.0 ± 0.4

**Table 2:** The values of the tumor growth inhibition index (TGII) for cisplatin and nanodrug receiver mice on various days after tumor cell inoculation.

Animal Group	Days after Tumor Cell Inoculation			
	10	18	26	35
**Cisplatin**	2.6 ± 0.5	19.0 ± 1.9	19.4 ± 2.0	31.0 ± 2.5
**Nanodrug**	8.0 ± 0.9	41.0 ± 3.0	35.0 ± 2.0	40.0 ±3.0

## Abbreviations

PBCA	Polybutylcyanoacrylate
NP	Nanoparticle
MDR	Multidrug resistance
PEG	Polyethylene glycol
MTT	3-(4,5-dimethylthiazol-2-yl)-2,5-diphenyltetrazolium bromide
AAS	Atomic absorption spectroscopy
H&E	Hematoxylin and eosin staining
BCA	Butylcyanoacrylate
PBS	Phosphate-buffered saline
PCS	Photon correlation spectroscopy
SEM	Scanning electron microscopy
FBS	Fetal bovine serum
IC_50_	Half maximal inhibitory concentration
TGII	Tumor growth inhibition index
ATN	Acute tubular necrosis
DNA	Deoxyribonucleic acid
